# Offspring fertility and grandchild survival enhanced by maternal grandmothers in a pre-industrial human society

**DOI:** 10.1038/s41598-021-83353-3

**Published:** 2021-02-11

**Authors:** Simon N. Chapman, Mirkka Lahdenperä, Jenni E. Pettay, Robert F. Lynch, Virpi Lummaa

**Affiliations:** 1 Department of Biology, University of Turku, Turku, Finland; 2 Department of Social Research, University of Turku, Turku, Finland; 3 Department of Public Health, University of Turku and Turku University Hospital, Turku, Finland; 4 Department of Anthropology, Pennsylvania State University, State College, USA

**Keywords:** Evolutionary theory, Social evolution

## Abstract

Help is directed towards kin in many cooperative species, but its nature and intensity can vary by context. Humans are one of few species in which grandmothers invest in grandchildren, and this may have served as an important driver of our unusual life history. But helping behaviour is hardly uniform, and insight into the importance of grandmothering in human evolution depends on understanding the contextual expression of helping benefits. Here, we use an eighteenth-nineteenth century pre-industrial genealogical dataset from Finland to investigate whether maternal or paternal grandmother presence (lineage relative to focal individuals) differentially affects two key fitness outcomes of descendants: fertility and survival. We found grandmother presence shortened spacing between births, particularly at younger mother ages and earlier birth orders. Maternal grandmother presence increased the likelihood of focal grandchild survival, regardless of whether grandmothers had grandchildren only through daughters, sons, or both. In contrast, paternal grandmother presence was not associated with descendants’ fertility or survival. We discuss these results in terms of current hypotheses for lineage differences in helping outcomes.

## Introduction

One common explanation for helping behaviour is kin selection, whereby an individual gains fitness benefits by helping relatives^1^. Grandmothers are among a class of kin whose help is known to improve offspring outcomes, but this is taxonomically rare^2–6^ partly because few species have an extended post-reproductive life^7,8^—though post-reproductive life is not necessarily a prerequisite for grandmothering^2^—and partly due to dispersal patterns not facilitating grandmothering^9^. In humans, grandmothers routinely invest in their grandchildren^10,11^, which is associated with increased survival^12–14^, improved nutrition^15,16^, and also contributes to their well-being^10^. However, although most studies to-date have concentrated on grandchild outcomes, the fitness benefits of grandmothering are two-fold: not only do grandmothers increase the likelihood that their grandchildren will survive, they also relieve their children of substantial parental investment duties, which may allow them to shorten inter-birth intervals and ultimately produce more grandchildren^10,13,15,17^. These two types of beneficial effects form the basis of the grandmother hypothesis, which suggests grandmothering can lead to selection favouring longer post-reproductive lifespans^13,15^.

Even when potential helpers have the same degree of relatedness (i.e. kinship coefficient) to dependent offspring, the costs and benefits of help can vary between individuals, leading to differential levels of help^18–20^. For example, mothers generally provision more than fathers^21–23^, with paternal care more likely to rely on context (i.e. changing depending on behavioural or environmental cues)^24–27^. Dispersal patterns are crucial in determining the access of kin, and human dispersal in particular is flexible and diverse. As the strength of selection on post-reproductive lifespan can be affected by whether one or both lineages provide benefits, it is necessary to study lineage differences (i.e. maternal or paternal) in outcomes from grandmother presence in societies where both maternal and paternal kin have the opportunity to help.

For survival outcomes, there is substantial evidence that maternal and paternal grandmothers do not always invest equally, with care typically exhibiting a matrilineal bias (i.e. the mother’s mother tends to invest more)^14,28–33^. In some societies, paternal grandmothers have even been found to be detrimental to the survival^14,28,34,35^ or physical development^32^ of their grandchildren. However, this is hardly universal: maternal grandmothers are not always beneficial to grandchildren^36^, and paternal grandmothers can both invest more than maternal grandmothers^37^ and are associated with increased grandchild survival^38,39^. Nor is there mutual exclusivity in being a maternal or paternal grandmother, because one can have grandchildren through both sons (paternal) and daughters (maternal). Might differences seen between the lineages be driven by preferential investment, when the choice is available? Alternatively, could grandmothers make a conscious effort to be ‘fair’ in their investment, and therefore be associated with better survival outcomes for paternal grandchildren only when they also have maternal grandchildren? To date, no work has explicitly investigated whether the presence of grandmothers with grandchildren from both sons and daughters leads to different outcomes compared to those with grandchildren from only one lineage.

In contrast to grandchild survival outcomes, potential lineage differences in the effects of grandmother presence of the fertility of the next generation (i.e. daughters and daughters-in-law) are less well established, with relatively few studies distinguishing between the fertility impacts of (grand)mothers and (grand)mothers-in-law^11^, and fewer still within the same models (allowing comparison of their effects). Increased fertility resulting from (grand)mother presence is an important component of the grandmother hypothesis^15^, and should therefore be considered in a holistic package alongside survival effects to identify all the effects of grandmothers in both lineages, with the eventual aim of evaluating the strength of natural selection on the length of post-reproductive lifespan in women in different contexts.

Here, we analyse the differential effects of grandmother lineages (maternal or paternal) in a high fertility pre-industrial Finnish population on a) the fertility of their daughters and daughters-in-law, and b) the survival of their grandchildren. In this society, dispersal was such that both grandmothers typically resided nearby, with paternal grandmothers often co-resident. Previous studies on this population have established grandmother effects on both offspring fertility and grandchild survival^13^, but these effects were not partitioned by lineage, and it remains unknown whether there are differential lineage effects in pre-industrial Finland. By distinguishing between all combinations of grandmothers (both lineages, maternal only, paternal only), we can directly compare the effects of maternal and paternal grandmother presence (separately and in combination) versus the effects of not having any living grandmother. First, we test for fertility effects of (grand)mother/(grand)mother-in-law presence on i) the probability and timing of first birth and ii) probabilities of subsequent births of grandoffspring and birth spacing. We used time-event models, as these analyses take into account age-specific fertility, which can potentially confound studies of this nature: grandmothers are more likely to be alive when their offspring are younger. Second, we test for differences in grandchild survival between lineages during childhood. Because grandmother effects are known to differ by the age of grandchildren^12,14,35,38^, we investigate differences in grandmother effects across three distinct phases of childhood (ages 0–2, 2–5, and 5–15). Third, we investigate whether the effects of grandmothers are influenced by the type of grandchildren available. In other words, whether these effects differ between cases when both types of grandchildren (i.e. maternal and paternal) are alive and cases when only maternal or only paternal grandchildren are alive. This is important to consider because being a maternal grandmother does not preclude one from being a paternal grandmother, yet to our knowledge this has not been accounted for before. All these analyses allow direct comparisons between lineages, and aid in clarifying how grandmother lineage may be a key factor in the strength of selection from grandmothering on post-reproductive lifespan.

## Methods

### Study population

We investigated how mother fertility and grandchild survival were affected by grandmother presence using an extensive pre-industrial demographic dataset collected from population registers (see e.g.^40,41^) for eight geographically-separated rural parishes in four regions of historical Finland (Southwest Finland: Rymättylä, Hiittinen, Kustavi; Pirkanmaa: Ikaalinen, Tyrvää; Northern Ostrobothnia: Pulkkila; Karelia: Rautu, Jaakkima) between 1731 and 1905. These registers—detailing births, deaths, children, occupations, and marriages—were maintained by the Lutheran Church, and were used to construct full life-histories of individuals and their descendants. Overall, Lutheranism was the prevalent religion, with 98% in the country still practicing in 1900^42^. Orthodox Christianity was confined mostly to Karelia, but still at a low percentage.

Finnish society in the eighteenth and nineteenth centuries was agrarian^43^ and largely pre-dates industrialisation^44^, which brought better living standards, better medicine and medical care, and birth control. Together, these factors contributed to decreases in birth rates and childhood mortality^45^ from the high levels typical of pre-demographic transition societies^46,47^. Adult life expectancy was greater than 60 years^41^, and an average grandmother and grandchild would live at the same time (and therefore could help and be helped, respectively) for less than 10 years across the study period^48^. Residence was typically patrilocal, and migration was constrained such that both sets of grandparents typically lived nearby and marital partners came from the same or nearby villages. We categorised individuals as belonging to one of the two following social classes, which can capture important between-individual variation in mortality, marriage patterns, and birth rate^49^: landed (e.g. farm owners, merchants) and landless (e.g. tenant farmers, fishermen, craftsmen, labourers, servants, crofters). Social stratification was, however, less pronounced than elsewhere in Europe, and dispersal to urban areas (thus increasing geographic distances between generations) did not occur until the late nineteenth century^50^.

Here, we included all individuals who were born between 1731 and 1890 and whose two grandmothers were both known (n = 5815 children; 1034 maternal grandmothers and 1003 paternal grandmothers, of which 298 grandmothers were both maternal and paternal), and their mothers, for the fertility analyses (see below for analysis-specific sample sizes).

### Statistical analysis

All analysis was conducted with R 3.5.1^51^. Significance was defined at the level of α = 0.05. Model selection was done by removing each fixed term with the function drop1, and then comparing their Akaike information criterion (AIC) values to those of the full model. To avoid overfitting—see^52^ for further details—terms were not included in the final model if AIC decreased upon removal, or if it was within 2 units of the full model (more conservative than higher thresholds)^52^. We did not, however, remove mother/grandmother status or focal individual age, as the former was the main term of interest, and the latter is a necessary component in our modelling structure (see below for details).

All analyses were discrete time-event analyses, which allowed us to analyse the effects of time-dependent variables on a yearly basis. These were implemented as binomial generalised linear mixed-effects models (GLMMs) using glmer from the R package lme4^53^, with the logit link function. Statistical significance of interaction terms was assessed via likelihood ratio testing, implemented with the mixed function in R package afex 0.21–2^54^.

#### Grandmother effects on fertility

##### Age at first reproduction

The age at which reproduction begins can be of greater importance for determining the length of an individual’s reproductive career than the less variable age of reproductive cessation^55^. Whilst women in this population did not co-reside pre-maritally with the paternal grandmother (their future mother-in-law), the paternal grandmother could still potentially accelerate the onset of reproduction by influencing who and when her son married. Therefore, we included women from the age of 16 (the end of childhood) until they gave birth or reached age 50 (n = 1560, n_observations_ = 17,411; oldest age at first birth was 44). In addition, we only included women who were ever married and had known information on their mother-in-law. As spousal death would disrupt reproductive scheduling, women were censored in the year their first spouse died.

The response variable was whether the offspring had their first birth in that year (0 = no birth, 1 = birth). The main explanatory variable was the interaction of age (mean-centred) and mother/mother-in-law survival status (4-level factor: only mother alive, only mother-in-law alive, both alive, neither alive), with ‘neither alive’ as the reference level, to test whether the presence of either mother or mother-in-law enhanced reproduction at age stage of the woman’s potential reproductive career. For any given single individual, mother/mother-in-law presence could change from year to year, depending on when the preceding generation died.

We initially controlled for social class, region, birth order, quadratic (mean-centred) age, and number of living siblings, while entering mother ID as a random effect to account for shared family effects between siblings. We did not control for birth cohort in the final model, because age at first birth did not vary across time in this population^48^ (though running the model with this random term included did not affect the results). After model selection, we dropped the social class and region terms.

##### Probability of subsequent births and birth spacing effects

Birth spacing is a crucial factor in determining an individual’s reproductive history. To analyse the effect of mother/mother-in-law presence both on probability of subsequent births and birth spacing in the middle generation, we restricted our analysis to a subset of women who had birthed more than one child (n = 1149, n_observations_ = 25,161) starting from after their first child’s birth.

The response variable was whether the individual gave birth in a given year (binary; 1 = birth, 0 = no birth). We included two three-way interactions as explanatory variables. The first was between a time-varying mother/mother-in-law presence factor, time since last birth (continuous; mean-centred), and age (mean-centred), and the second between time-varying mother/mother-in-law presence variable, time since last birth, and birth order of the grandchild. Time since last birth was used instead of inter-birth interval, as the latter term is time invariant and inappropriate for this type of model. This birth order term was for the child born at the end of the interval (e.g. all years between the births of child two and child three, including the birth year of child three, would be given birth order of 3), and was included as parity could affect desire to have more children. After the last birth, subsequent years had birth order coded as the next value (e.g. if the fourth child was the last one born to an individual, the remaining years would have this term set as 5). All two-way combinations from these interaction terms were also added to the model. We also included a two-way interaction of the survival status of the child born at the start of the interval (binary: alive 1, dead 0) and the time since last birth, as this can affect the speed and probability of giving birth (Sear et al. 2003). Rather than removing observations in which the time since last birth exceeded 10 years^56^, we capped longer inter-birth intervals at 10 years in order to prevent these extreme cases from exerting undue influence on the results. We controlled for birth order of the focal individual, region of Finland, number of living siblings of the focal individual, and social class. Birth cohort of their first-born child (16-level factor, with ten year bins e.g. 1731–1740 etc.) was entered as an additional random effect to account for an uneven spread of data and differences in conditions (social and environmental) across the period of study that may affect inter-birth intervals. Using the results of our model selection criteria (see above), we removed the birth order of the focal individual and number of living siblings terms.

#### Grandchild survival outcomes

In the survival analyses, the response variable was whether the focal grandchild survived in a given year (binary: alive 1, dead 0). Individuals without a recorded date of death and those with either grandmother disappearing from the records before they themselves reached the upper age limit (see below for details) or died were censored at the last date they were known to be alive. We excluded observation years for individuals from the analysis if the individual died within a week of the death of their mother (suggestive of high dependency or disease, and therefore unlikely to be prevented by grandmother intervention), or if the mother and child were both censored in that year (suggestive of a family level event e.g. dispersal to elsewhere in Finland, with the individuals awaiting follow-up by genealogists).

Grandmother presence was coded in a similar way to the fertility analyses above: as a 4-level factor with levels for both grandmothers alive, no living grandmother, only maternal grandmother alive, and only paternal grandmother alive. For a given individual, grandmother presence could change from year to year, depending on when their grandmothers died—this was only one directional e.g. once a maternal grandmother had died, the focal individual could not have ‘both alive’ or ‘maternal grandmother only’ as the value for grandmother presence. As precise housing information is unavailable, grandmother distance to a grandchild was considered at the parish level by comparing the last known parish of a grandmother to the birth parish of a grandchild. All grandmothers who were both coded as “alive” and included in the analysis needed to have lived in the same parish at the same time as the grandchild. Individuals with one or both grandmothers alive but living in a different parish were not included in the sample, as these grandmothers could not be treated as either present or as dead. Furthermore, grandmothering effects have been shown to decline with distance^57^, so including grandmothers living in a different parish could mask the effects of nearby grandmothers.

##### Grandchild survival at different childhood stages

To investigate how maternal and paternal grandmother presence was associated with grandchild survival at different grandchild ages, we separated the data into three age classes: 0–2 (when the child is breast-fed; n = 5784, n_observations_ = 11,013), 2–5 (n = 4823, n_observations_ = 13,811), and 5–15 (when child mortality greatly declines—only 20% of childhood deaths occur after age 5 ; n = 4299, n_observations_ = 43,672). These age intervals include the left border and exclude the right border, with the exception of 5–15, which included all individuals up to and including the age of 15.

In addition to the grandmother presence term, the initial full models also contained terms for child age (continuous; linear and quadratic), maternal survival status (alive, dead, unknown status), childhood social class (2-level factor: landed, landless), whether the child was a twin, sex of grandchild, birth order, region of Finland, maternal age (continuous), and number of surviving siblings (continuous, controlling for within-family competition). Of these, grandmother presence, child age (linear and quadratic), maternal survival status, and number of living siblings were time-varying. Random terms included mother identity (ID) nested within maternal grandmother ID to account for variation between groups of siblings (from mother ID) and cousins (from grandmother ID), and birth cohort.

Using the model selection procedure outlined above, quadratic age, birth order and mother age at birth were removed from all three models. Additionally, childhood social class, region, and sex were removed from 2 to 5 and 5 to 15 models, number of living siblings was removed from 0 to 2 and 2 to 5 models, and twin was removed from the 5–15 model. These new, more parsimonious models were run with the grandmother type ‘no living grandmother’ as the reference level, which provided the intercept values. Reference levels for other variables were as follows: ‘Southwest Finland’ for region, ‘mother alive’ for mother survival status, and ‘singleton’ for twin.

##### Grandchild survival by lineage exclusivity

To investigate whether the presence of grandmothers who have both maternal and paternal grandchildren are correlated with differing survival outcomes in maternal and paternal grandchildren compared to those with only maternal or only paternal grandchildren, we again ran GLMMs on grandchildren aged 0–2 (n = 5784, n_observations_ = 11,013), 2–5 (n = 4823, n_observations_ = 13,811), and 5–15 (n = 4299, n_observations_ = 43,672). The grandmother type variable was updated to account for whether maternal grandmothers also had paternal grandchildren, and whether paternal grandmothers also had maternal grandchildren. The new variable therefore had six levels: both grandmothers alive (observations: 0–2 n = 3743; 2–5 n = 3896; 5–15 n = 6835), both grandmothers dead (observations: 0–2 n = 2307; 2–5 n = 3535; 5–15 n = 18,578), maternal grandmother only (observations: 0–2 n = 2245; 2–5 n = 2807; 5–15 n = 7702), paternal grandmother only (observations: 0–2 n = 1702; 2–5 n = 2134; 5–15 n = 5780), maternal grandmother of the child but also a paternal grandmother (observations: 0–2 n = 575; 2–5 n = 826; 5–15 n = 2602), and paternal grandmother of the child but also a maternal grandmother (observations: 0–2 n = 441; 2–5 n = 613; 5–15 n = 2175). Due to separation issues (i.e. non-existence/exceedingly low sample size of certain combinations of variables), we were unable to account for whether grandmothers were maternal and paternal when both grandmothers were alive.

Fixed and random terms were the same as in the previous analysis, and we used the same model selection procedure as previously. The terms retained in the final models were as follows: for 0–2, grandmother type, age, twin status, childhood social class, sex, maternal survival status, and region; for 2–5, grandmother type, age, and maternal survival status; and for 5–15, grandmother type, age, number of living siblings, and maternal survival status.

## Results

We preface the results with the caveats that all analyses are correlative and that no direct measures of investment (e.g. contact time) are present in historical datasets such as ours.

### Lineage-specific (grand)mother effects on offspring fertility

#### Age at first reproduction

**Figure 1:**
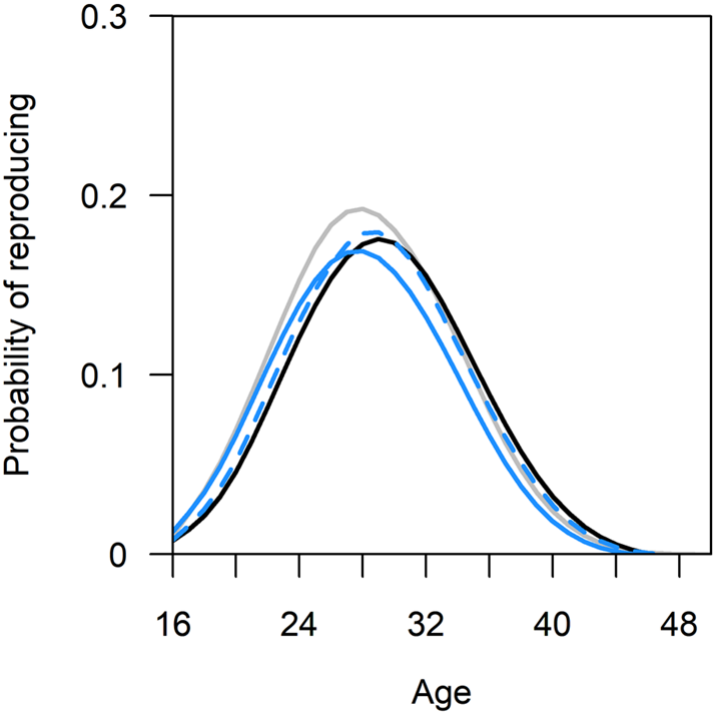
Age-specific probability of reproducing for the first time by presence of mother or mother-in-law. Smoothing splines fitted from model-predicted reproduction probabilities using smooth.spline function. Grey line = neither mother nor mother-in-law alive, black = both alive, solid blue = mother alive, dashed blue = mother-in-law alive. This model controlled for birth order and number of living siblings.

Descriptive statistics of the data show that the mean age at first reproduction was earliest when both the mother and mother-in-law were alive (24.00 ± 3.96), and latest when both were deceased (26.75 ± 5.40). If only the mother was living, first reproduction of daughters was slightly earlier than if only the mother-in-law was alive (24.80 ± 4.75 vs. 25.26 ± 4.62). However, the mother/mother-in-law presence did not have a statistically significant effect on the probability of the first birth overall (Table S1), or at different ages of offspring, as the interaction of age and mother/mother-in-law presence was not statistically significant (χ_3_^2^ = 7.25, p = 0.06). In other words, age at first reproduction was not earlier in the presence of the preceding generation (Fig. 1).

#### Probability of subsequent births and birth spacing effects

**Figure 2:**
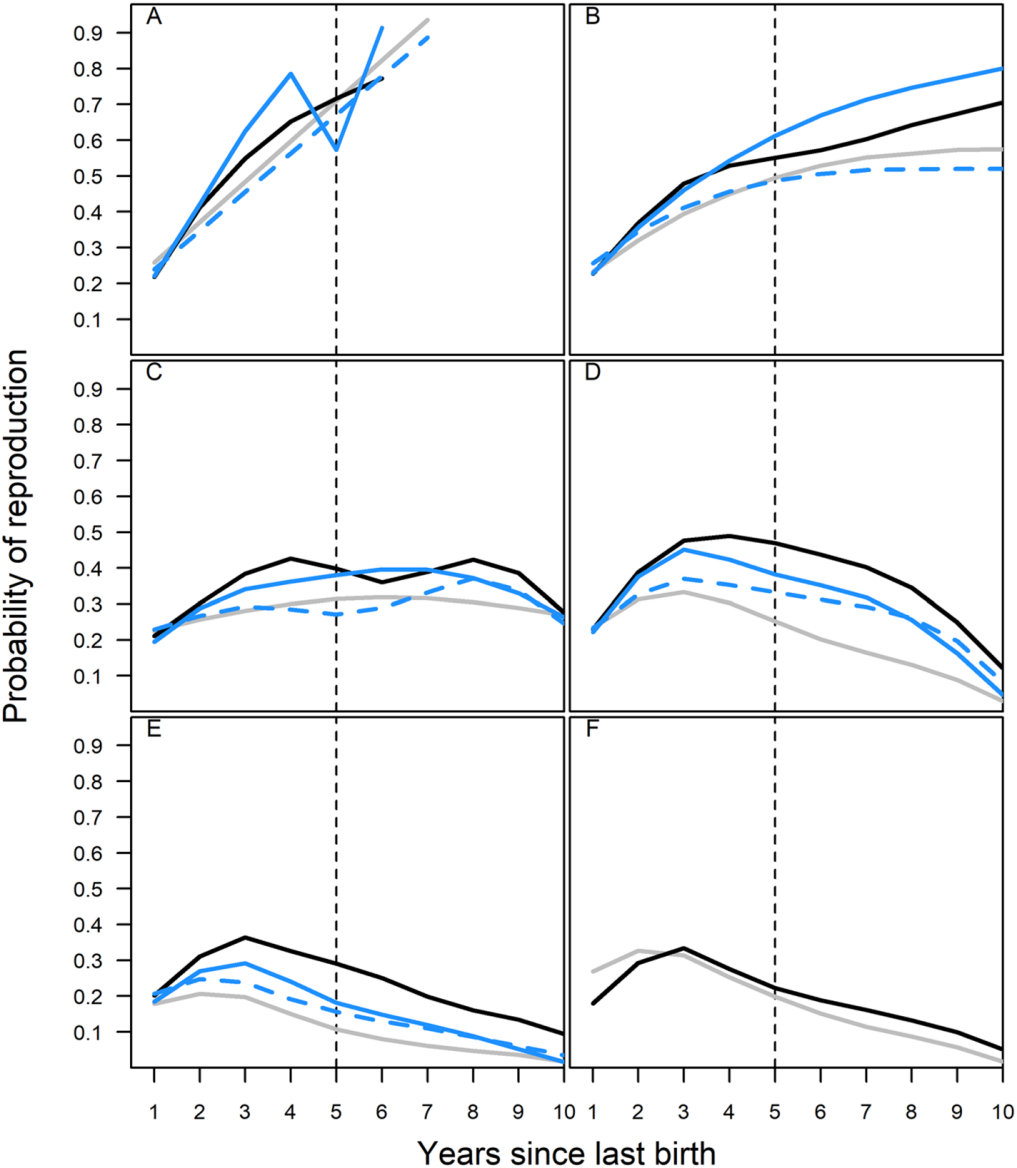
Mother presence and inter-birth interval effects on subsequent reproduction at different ages (**A**–**C**) and birth orders (**D**–**E**). Panels: (**A**) Mother/mother-in-law presence at age 25; (**B**) mother/mother-in-law presence at age 30; (**C**) mother/mother-in-law presence at age 35; (**D**) mother/mother-in-law presence for earlier-born children; (**E**) mother/mother-in-law presence when child birth order was greater than 4; (**F**) survival of previous child. Line colour in panels (**A**–**E**) indicates presence of mother and mother-in-law: grey = both dead, black = both alive, solid blue = mother alive, dashed blue = mother-in-law alive. In panel (**F**), grey indicates previous child was dead, and black indicates they were still alive. Birth order of the child was continuous in the models, but has been grouped in panels (**D**) and (**E**) following^13^. Smoothing splines fitted from model-predicted reproduction probabilities using the smooth.spline function. Vertical dashed line at year 5 shows the age at which the previous child would have got past the ‘toddler’ stage of life, and predicted reproductive probability thereafter would be less meaningful in regards to the Grandmother Hypothesis.

We found that when a mother or both the mother and mother-in-law were alive it was associated with a positive effect on offspring probability of next birth. This effect depended, however, on offspring age and birth-order of the grand-offspring (Table S2). The probability of next birth for daughters was highest at earlier birth-orders than later birth-orders when the mother was alive, either solely or at the same time as the mother-in-law (Figure S1; Table S2), which would result in higher numbers of children during the lifetime of those daughters whose mothers were alive as compared to those whose mother was not living during their reproductive years. Furthermore, there were significant birth spacing effects associated with mother and both mother and mother-in-law presence on the probability of birth. We found a significant three-way interaction between time since last birth, age, and mother/mother-in-law status (χ^2^_3_ = 31.53, p < 0.001); when the mother was present, the probability of her daughter giving birth at shorter birth intervals was higher and the effect was stronger at younger ages (Fig. 2A–C). The shortening effect of mothers on their daughters’ birth intervals was also more pronounced for earlier birth orders (Fig. 2D,E): the 3-way interaction of time since last birth, mother/mother-in-law status, and birth order of the child was also significant (χ_3_^2^ = 15.27, p = 0.002; Table S2). In panels A–E of Fig. 2, the probability of next birth was higher when the mother was alive (either by herself or with the mother-in-law), suggesting that mother presence during her daughter’s reproductive lifespan may lead to more grandchildren than if she were absent.

### Lineage-specific grandmother effects on grandchild survival

#### Grandchild survival at different childhood stages

We found that, of the 5815 children included in our analyses, 31.4% (1826) died during childhood, and an additional 7.2% (421) were censored before 15. To parse the age-specific effects of maternal or paternal grandmother presence on such grandchild mortality (i.e. how sensitive grandchildren were to grandmother presence at different stages of development), we investigated the influence of grandmothers for key grandchild age periods. Grandchild mortality was greatest early in life, with 53.1% of childhood deaths occurring in those younger than two, and 79.9% of deaths occurring in children younger than five.

**Figure 3:**
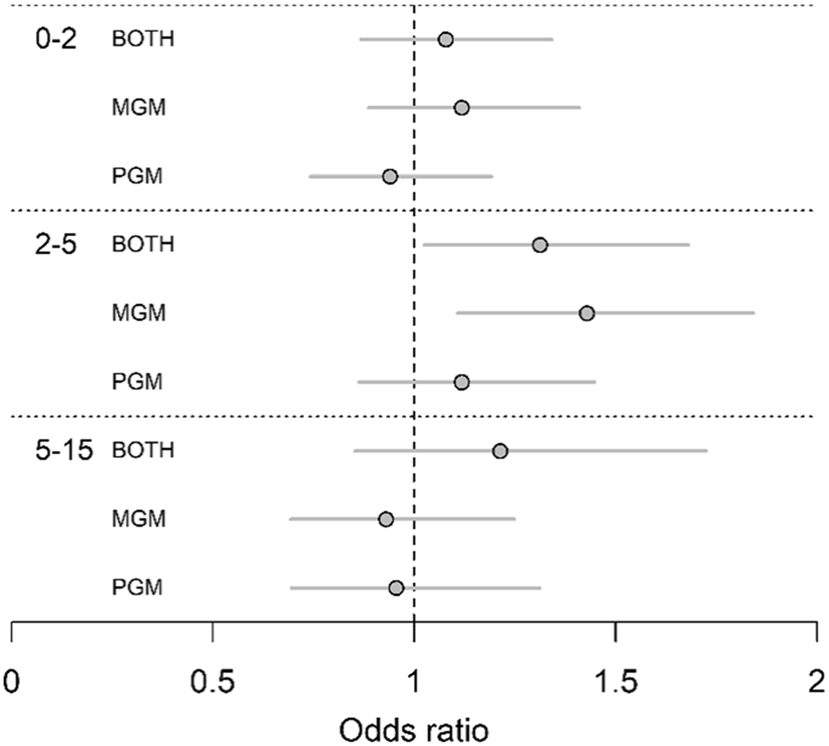
Odds ratios of grandchild survival by grandmother presence for each age class. *MGM* maternal grandmother alive, *PGM* paternal grandmother alive. Lines for each value are 95% confidence intervals. Vertical dashed line indicates an odds ratio of 1; an overlapping odds ratio indicates there is no meaningful difference in survival compared to the baseline of having no living grandmother. All models controlled for mother survival status. Age class 0–2 additionally controlled for sex, whether the focal individual was a twin, social class, and region of Finland, and age class 5–15 controlled for number of living siblings.

We found that grandmother effects varied between different age groups (Fig. 3; Table S3). Between ages 0–2, when children mostly depended on their mother for feeding, grandchild survival did not significantly differ between those without any living grandmothers and those with a living maternal grandmother only (β = 0.112 ± 0.118, p = 0.345; OR 1.118 [0.887, 1.409]), living paternal grandmother only (β = − 0.062 ± 0.121, p = 0.609; OR 0.940 [0.742–1.191]), or both grandmothers living (β = 0.075 ± 0.111, p = 0.500; OR 1.078 [0.867–1.340]). Between ages 2–5, however, there was a positive effect on survival associated with having both grandmothers alive compared to having no living grandmothers (β = 0.272 ± 0.126, p = 0.031; OR 1.312 [1.025, 1.680]). Similarly, having a maternal grandmother was associated with better survival than having no grandmother at all (β = 0.356 ± 0.130, p = 0.006; OR 1.428[1.107, 1.841]). Again, there was no statistically significant difference between having a paternal grandmother and having no grandmother (β = 0.111 ± 0.132, p = 0.399; OR 1.118 [0.863, 1.447]). From the age of five onwards, when overall mortality risk had greatly reduced compared to the early years, there were no statistically significant differences in survival outcomes of grandchildren between any of the grandmother types: grandmother presence or absence was not associated with survival of their grandchildren after early childhood (Table S3).

#### Grandchild survival by lineage exclusivity

**Figure 4:**
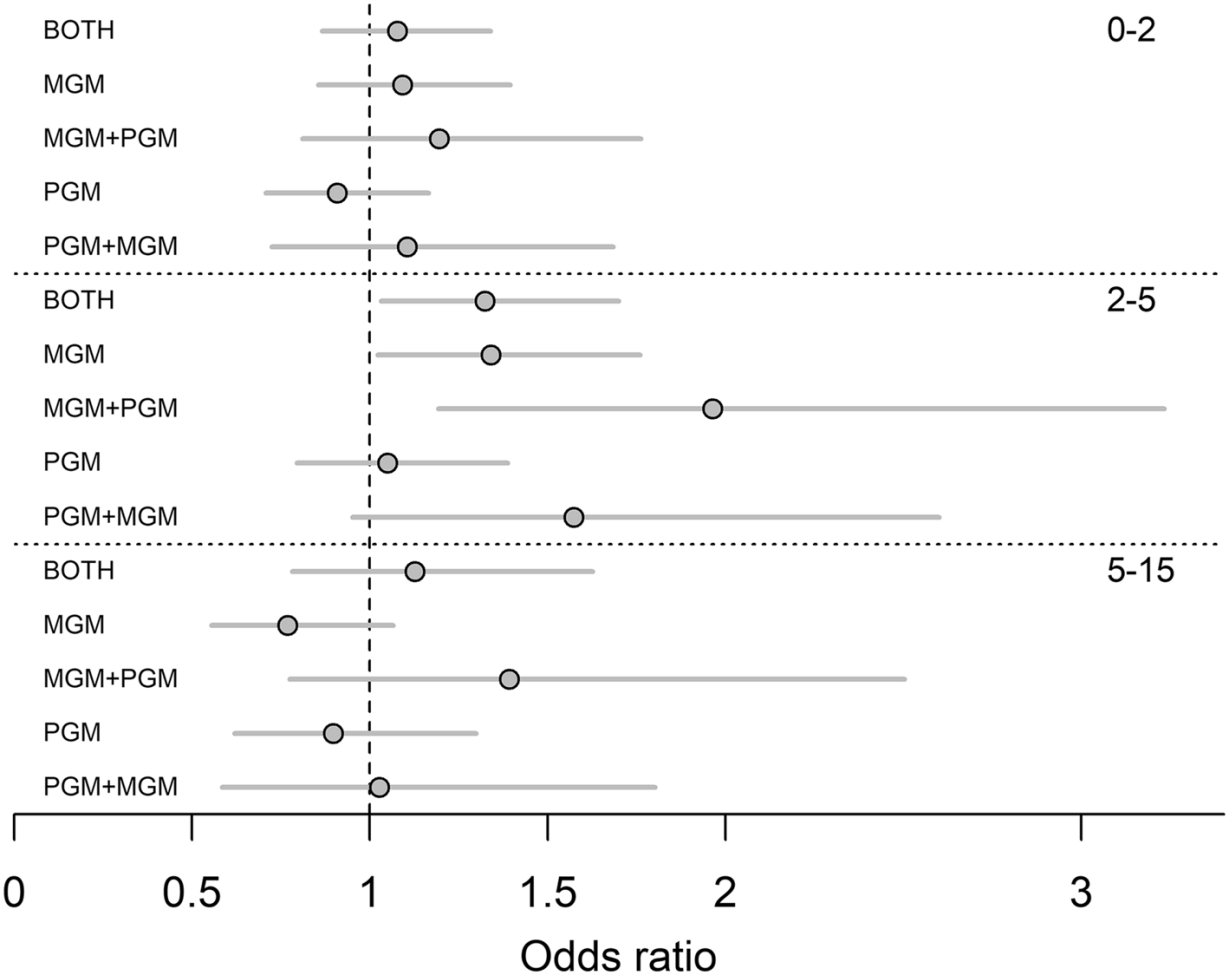
Odds ratios of grandchild survival by grandmother presence and lineage exclusivity of grandmothers. *MGM* maternal grandmother alive, *PGM* paternal grandmother alive, *MGM + PGM* maternal grandmother alive and has living grandchildren through son, *PGM + MGM*  paternal grandmother alive and has living grandchildren through daughter. Lines for each value are 95% confidence intervals. Vertical dashed line indicates an odds ratio of 1; an overlapping odds ratio indicates there is no meaningful difference in survival compared to the baseline of having no living grandmother. All models controlled for mother survival status. Age class 0–2 additionally controlled for sex, whether the focal individual was a twin, social class, and region of Finland, and age class 5–15 controlled for number of living siblings.

We then investigated whether lineage exclusivity of a grandmother (i.e. the grandmother had grandchildren through sons only, daughters only, or through sons and daughters) may have played a role in generating differences in grandchild outcomes by maternal and paternal grandmother presence. We found that there were no grandmother effects of any kind for infants (ages 0–2) or for older children (ages 5–15), whilst effects were found for ages 2–5 (Fig. 4; Table S4). Significant effects were only seen for maternal grandmothers, regardless of whether they only had grandchildren via daughters (β = 0.294 ± 0.139, p = 0.034; OR 1.34 [1.02, 1.76]), or whether they were also paternal grandmothers (β = 0.675 ± 0.254, p = 0.008; OR 1.96 [1.19, 3.23]). Grandmother presence was not correlated with a survival benefit to paternal grandchildren, regardless of whether these grandmothers had maternal grandchildren or not. However, the non-significant effect size of paternal grandmothers with maternal grandchildren was large (β = 0.453 ± 0.256, p = 0.080), suggesting that having grandchildren through sons and daughters could potentially bring some small benefit to the paternal grandchildren, given the right circumstances (e.g. resource-dependent).

## Discussion

We found a matrilineal bias in grandmothering outcomes such that both higher offspring fertility and grandchild survival were associated with the presence of a maternal grandmother. In particular, maternal grandmothers were associated with an increased probability of birth after the first child and with shorter birth intervals of their daughters, and did this more when they were younger and earlier in their reproductive career (i.e. earlier births). Overall, this acceleration of reproductive scheduling in the presence of maternal grandmothers after the onset of reproduction supports the grandmother hypothesis. However, effects did not exist for all outcomes or contexts: for fertility, (grand)mother presence did not influence age at first birth, and survival effects on grandchildren were limited to the period after they had been weaned, with no detectable effects across the entirety of childhood. This may be due to the comparatively low mortality rates after age 5 (only ~20% of childhood deaths occurred after this age): the effect of maternal grandmothers is higher with toddlers, reiterating the importance of considering grandchild age when investigating the fitness outcomes of helping kin. Regardless, these fertility results show that both facets of the grandmother hypothesis can occur at the same time in the same population: maternal grandmothers could positively affect fertility of offspring and survival of grandoffspring.

Paternity uncertainty is frequently raised as a possible explanation for results showing differences between maternal and paternal grandmother investment^10,31,39,58,59^. However, this interpretation cannot completely explain fertility differences, and is unlikely to explain the full effect of lineage differences in grandchild outcomes in this pre-industrial Finnish population. There are two elements of paternity uncertainty: psychological adaptations and a population parameter. The former invokes kin recognition and fidelity, which could reduce paternal grandparent attachment towards paternal grandchildren. In this population, these psychological adaptations are unlikely to explain lineage differences: monogamy was strictly adhered to in this population, with the church forbidding divorce and enforcing punishment for adultery^60^ (thus the social environment provided fathers with little reason to doubt paternity). Indeed, historical extra-marital paternity rates in Europe are estimated to have been very low during this period (less than 1.5%)^61,62^. The population parameter of paternity uncertainty is an ancestral, taxon-wide parameter, but the impact this might have had on patrilineal grandmaternal behaviour is unknown and may be negligible. Furthermore, paternity uncertainty as an explanation often ignores societal differences^10,63^, is not universally supported^64,65^, and cannot be explicitly tested in historical societies without genotyping. Differential X-chromosome inheritance (i.e. paternal grandmothers are on average more related to granddaughters via the X-chromosome than maternal grandmothers are) has also been proposed as a possible mechanism for lineage differences in optimal grandmother investment strategies^66^, but this too has been found to be inapplicable to this population^46^. Similarly, whilst localised resource competition^67^ could explain differences in cases with negative effects of resident grandmothers on grandchild outcomes^35^ (e.g. limited food resources would be shared between more people in three-generational households), they cannot explain differences when the co-resident lineage has no impact on survival, as is the case here. Perry and Daly^68^ raise the intriguing possibility that investment in the grandchildren is also a downstream investment in the mother’s ability to help others. Though this work outlines testable hypotheses, the authors themselves acknowledge the difficulties in addressing these without measures of alloparental contributions (one of the downsides of using historical register data).

Studies into differential ‘helping’ outcomes (though of course these are correlational and do not directly measure help) could be confounded by maternal grandmothers also being paternal (and vice versa). However, we found no evidence that lineage ‘exclusivity’ drives the difference in grandmother effect. Grandmothers with grandchildren from both daughters and sons were just as beneficial to their daughter’s children at grandchild ages 2–5 as grandmothers with grandchildren only through their daughters (i.e. exclusively maternal). Meanwhile, paternal grandmothers without grandchildren through their daughters did not improve the survival of grandchildren, which suggests that competition between daughters and sons was not a reason for the failure of paternal grandmothers to improve the fitness outcomes of their children. These results do not support preferential investment or any kind of ‘fair’ allocation of help, as paternal grandmothers remain unassociated with survival whether they have the option to invest in maternal grandchildren or not. Grandmother presence does not improve survival of their paternal grandchildren—something must be driving these clear lineage differences in survival outcomes, but we have not been able to identify it here.

Regardless of the exact driver of lineage differences in grandmother effects, current hypotheses do not have to be mutually exclusive. Indeed, cultural practices and societal structure (e.g. dispersal and residency patterns, subsistence strategy, religious practices etc.) may be the most important mediators of differential grandmaternal investment by altering the relative contributions of the various (potential) mechanisms (see e.g.^68^). The effect of culture is difficult to empirically test, even accounting for dispersal and co-residence patterns, and makes it more challenging to determine the exact role of grandmothering in selection for post-reproductive lifespan as we delve further back into our evolutionary past. From an even broader perspective, however, one cannot look at any single factor as a key predictor of lineage-specific grandmothering. For example, though female-biased dispersal could predispose species to evolve extended post-reproductive lifespans and post-reproductive help^7,9,15^, it does not alone allow prediction of how grandmother effects are expressed. For example, even though pre-industrial Finland and rural Gambia share broad societal similarities—the populations are typically agrarian and tend towards patrilocal living—the fertility effects are reversed. In Gambia, it is the paternal grandmother (mother-in-law) that is associated with improved fertility in Gambia^69^, whereas in Finland the maternal grandmother increases it. Accounting for more societal and cultural elements reveals the populations are only superficially similar and differ in e.g. mating strategy and practised religion, emphasising the importance of considering multiple criteria for ‘defining’ societies.

In conclusion, we have found maternal grandmother presence correlates with improved outcomes for mother fertility and the survival of maternal grandchildren. The widespread findings of a benefit from one or other (but rarely both) grandmaternal lineages suggest that selection on post-reproductive lifespan from grandmothering may be weaker than once thought. As mechanisms currently hypothesised as drivers of lineage differences do not appear to produce the different effects found in this population, it may be that a suite of cultural practices mediate lineage biases in outcomes (and likely grandmother investment).

## Electronic supplementary material

Below is the link to the electronic supplementary material.

Supplementary Information 1.

Supplementary Information 2.

Supplementary Information 3.

Supplementary Information 4.
