# Supplementary material for: Offspring fertility and grandchild survival enhanced by maternal grandmothers in a pre-industrial human society
Source: Sci Rep. 2021 Feb 11;11:3652. doi: 10.1038/s41598-021-83353-3 (PMC7878921; doi:10.1038/s41598-021-83353-3)
Supplement: Supplementary file 1 [file 41598_2021_83353_MOESM2_ESM.odt]

Offspring fertility and grandchild survival enhanced by maternal grandmothers in a pre-industrial society 

Simon N. Chapman, Mirkka Lahdenperä, Jenni E. Pettay, Robert F. Lynch, Virpi Lummaa

Supplementary Tables

Table S1. Generalised linear mixed-effects models for age at first birth. MIL=mother-in-law, * denotes interaction. See methods for reference levels of variables.

|  | Effects | Estimate | Standard Error | z value | p |
| --- | --- | --- | --- | --- | --- |
| Fixed | Intercept | -1.720 | 0.136 | -12.64 | <0.001 |
|  | Relation (mother) | -0.087 | 0.139 | -0.63 | 0.532 |
|  | Relation (MIL) | -0.248 | 0.144 | -1.73 | 0.084 |
|  | Relation (both) | -0.327 | 0.129 | -2.54 | 0.011 |
|  | Age | 1.601 | 0.159 | 10.06 | <0.001 |
|  | Age (quadratic) | -0.848 | 0.048 | -17.58 | <0.001 |
|  | Birth order | 0.006 | 0.015 | 0.37 | 0.714 |
|  | Living siblings | -0.016 | 0.019 | -0.84 | 0.399 |
|  | Age * relation (mother) | -0.124 | 0.148 | -0.84 | 0.401 |
|  | Age * relation (MIL) | 0.143 | 0.155 | 0.93 | 0.354 |
|  | Age * relation (both) | 0.184 | 0.142 | 1.29 | 0.196 |
|  |  | Variance | Standard Deviation |  |  |
| Random | Mother ID | 0.473 | 0.689 |  |  |

Table S2. Generalised linear mixed-effects models for birth spacing effects on probability of birth. MIL=mother-in-law, TLB=time since last birth, * denotes interaction. See methods for reference levels of variables.

|  | Effects | Estimate | Standard Error | z value | p |
| --- | --- | --- | --- | --- | --- |
| Fixed | Intercept | -0.363 | 0.177 | -2.06 | 0.040 |
|  | Relation (mother) | -0.005 | 0.227 | -0.02 | 0.984 |
|  | Relation (MIL) | 0.193 | 0.230 | 0.84 | 0.402 |
|  | Relation (both) | 0.047 | 0.260 | 0.18 | 0.857 |
|  | Age | -0.171 | 0.011 | -15.02 | <0.001 |
|  | TLB | -0.212 | 0.050 | -4.21 | <0.001 |
|  | Birth order of next child1 | -0.102 | 0.030 | -3.44 | <0.001 |
|  | Survival of previous child | -0.019 | 0.054 | -0.35 | 0.729 |
|  | TLB * survival of previous child | 0.225 | 0.022 | 10.37 | <0.001 |
|  | TLB * birth order of next child | 0.059 | 0.009 | 6.86 | <0.001 |
|  | TLB * relation (mother) | -0.075 | 0.076 | -0.98 | 0.326 |
|  | TLB * relation (MIL) | 0.061 | 0.076 | 0.80 | 0.424 |
|  | TLB * relation (both) | -0.174 | 0.087 | 1.99 | 0.046 |
|  | Age * TLB | -0.068 | 0.004 | -16.99 | <0.001 |
|  | Age * relation (mother) | -0.046 | 0.017 | -2.67 | 0.008 |
|  | Age * relation (MIL) | 0.029 | 0.017 | 1.71 | 0.087 |
|  | Age * relation (both) | -0.005 | 0.019 | -0.27 | 0.784 |
|  | Relation (mother) * birth order of next child | 0.077 | 0.039 | 1.98 | 0.048 |
|  | Relation (MIL) * birth order of next child | -0.018 | 0.041 | -0.44 | 0.660 |
|  | Relation (both) * birth order of next child | 0.070 | 0.046 | 1.54 | 0.123 |
|  | Age * TLB * relation (mother) | -0.021 | 0.006 | -3.42 | 0.001 |
|  | Age * TLB * relation (MIL) | 0.010 | 0.006 | 1.56 | 0.118 |
|  | Age * TLB * relation (both) | -0.021 | 0.007 | -3.14 | 0.002 |
|  | TLB * relation (mother) * birth order of next child | 0.032 | 0.014 | 2.35 | 0.019 |
|  | TLB * relation (MIL) * birth order of next child | -0.011 | 0.014 | -0.76 | 0.446 |
|  | TLB * relation (both) * birth order of next child | 0.044 | 0.016 | 2.75 | 0.006 |
|  |  | Variance | Standard Deviation |  |  |
| Random | Mother ID | 0.134 | 0.366 |  |  |
|  | Mother ID:Focal ID | 0.872 | 0.934 |  |  |
|  | Birth cohort | <0.001 | <0.001 |  |  |

1See methods

Table S3. Generalised linear mixed-effects models for grandchild survival at different stages of childhood. MGM=maternal grandmother, PGM=paternal grandmother, GM type=grandmother type. See methods for reference levels of variables. Square brackets for odds ratios show lower and upper confidence intervals.

|  | Age range | Effects | Estimate | Standard Error | z value | p | Odds ratios |
| --- | --- | --- | --- | --- | --- | --- | --- |
| Fixed | 0-2 | Intercept | 3.184 | 0.249 | 12.78 | <0.001 |  |
|  |  | GM type (MGM) | 0.112 | 0.118 | 0.94 | 0.345 | 1.12[0.89,1.41] |
|  |  | GM type (PGM) | -0.062 | 0.121 | -0.51 | 0.609 | 0.94[0.74,1.19] |
|  |  | GM type (both) | 0.075 | 0.111 | 0.67 | 0.500 | 1.08[0.87,1.34] |
|  |  | Twin | -1.154 | 0.157 | -7.33 | <0.001 |  |
|  |  | Sex (female) | 0.199 | 0.072 | 2.76 | 0.006 |  |
|  |  | Age | 0.078 | 0.071 | 1.20 | 0.272 |  |
|  |  | Social class (landless) | 0.186 | 0.088 | 2.12 | 0.034 |  |
|  |  | Mother status (dead) | -2.235 | 0.207 | -10.79 | <0.001 |  |
|  |  | Mother status (censored) | 0.687 | 1.145 | 0.60 | 0.549 |  |
|  |  | Region (Pirkanmaa) | 0.100 | 0.107 | 0.93 | 0.351 |  |
|  |  | Region (Karelia) | -1.284 | 0.423 | -3.04 | 0.002 |  |
|  |  | Region (Northern Ostrobothnia) | 0.070 | 0.126 | 0.56 | 0.576 |  |
|  | 2-5 | Intercept | 2.112 | 0.209 | 10.13 | <0.001 |  |
|  |  | GM type (MGM) | 0.356 | 0.130 | 2.75 | 0.006 | 1.42[1.11,1.84] |
|  |  | GM type (PGM) | 0.111 | 0.132 | 0.84 | 0.399 | 1.12[0.86,1.45 |
|  |  | GM type (both) | 0.272 | 0.126 | 2.16 | 0.031 | 1.31[1.03,1.68] |
|  |  | Age | 0.379 | 0.057 | 6.62 | <0.001 |  |
|  |  | Mother status (dead) | -0.975 | 0.201 | -4.85 | <0.001 |  |
|  |  | Mother status (censored) | 0.188 | 0.723 | 0.26 | 0.795 |  |
|  | 5-15 | Intercept | 4.168 | 0.282 | 14.78 | <0.001 |  |
|  |  | GM type (MGM) | -0.072 | 0.150 | -0.48 | 0.630 | 0.93[0.69,1.25] |
|  |  | GM type (PGM) | -0.046 | 0.162 | -0.29 | 0.775 | 0.96[0.70,1.31] |
|  |  | GM type (both) | 0.193 | 0.179 | 1.08 | 0.282 | 1.21[0.85,1.72] |
|  |  | Age | 0.131 | 0.019 | 7.01 | <0.001 |  |
|  |  | Living siblings | -0.119 | 0.040 | -3.00 | 0.003 |  |
|  |  | Mother status (dead) | -0.280 | 0.162 | -1.73 | 0.083 |  |
|  |  | Mother status (censored) | 0.218 | 0.744 | 0.29 | 0.770 |  |
|  |  |  | Variance | Standard Deviation |  |  |  |
| Random | 0-2 | MGM ID:Mother ID | 0.200 | 0.447 |  |  |  |
|  |  | MGM ID | 0.207 | 0.455 |  |  |  |
|  |  | Birth cohort | 0.032 | 0.179 |  |  |  |
|  | 2-5 | MGM ID:Mother ID | 0.173 | 0.416 |  |  |  |
|  |  | MGM ID | <0.001 | <0.001 |  |  |  |
|  |  | Birth cohort | 0.071 | 0.267 |  |  |  |
|  | 5-15 | MGM ID:Mother ID | 0.504 | 0.710 |  |  |  |
|  |  | MGM ID | 0.204 | 0.451 |  |  |  |
|  |  | Birth cohort | 0.026 | 0.160 |  |  |  |

Table S4. Generalised linear mixed-effects models for grandchild survival at different stages of childhood by exclusivity of grandmother lineage. MGM=maternal grandmother, PGM=paternal grandmother, GM type=grandmother type. See methods for reference levels of variables. Square brackets for odds ratios show lower and upper confidence intervals.

|  | Age range | Effects | Estimate | Standard Error | z value | p | Odds ratios |
| --- | --- | --- | --- | --- | --- | --- | --- |
| Fixed | 0-2 | Intercept | 3.181 | 0.249 | 12.77 | <0.001 |  |
|  |  | GM type (MGM) | 0.088 | 0.125 | 0.71 | 0.480 | 1.09[0.86,1.39] |
|  |  | GM type (MGM.P) | 0.178 | 0.198 | 0.90 | 0.368 | 1.20[0.81,1.76] |
|  |  | GM type (PGM) | -0.097 | 0.127 | -0.76 | 0.447 | 0.91[0.71,1.17] |
|  |  | GM type (PGM.M) | 0.100 | 0.215 | 0.46 | 0.643 | 1.11[0.73,1.68] |
|  |  | GM type (both) | 0.074 | 0.111 | 0.67 | 0.506 | 1.08[0.87,1.34] |
|  |  | Twin | -1.151 | 0.158 | -7.31 | <0.001 |  |
|  |  | Sex (female) | 0.197 | 0.072 | 2.74 | 0.006 |  |
|  |  | Age | 0.075 | 0.071 | 1.06 | 0.287 |  |
|  |  | Social class (landless) | 0.193 | 0.088 | 2.19 | 0.028 |  |
|  |  | Mother status (dead) | -2.235 | 0.207 | -10.79 | <0.001 |  |
|  |  | Mother status (censored) | 0.665 | 1.132 | 0.59 | 0.557 |  |
|  |  | Region (Pirkanmaa) | 0.098 | 0.107 | 0.91 | 0.361 |  |
|  |  | Region (Karelia) | -1.245 | 0.424 | -2.94 | 0.003 |  |
|  |  | Region (Northern Ostrobothnia) | 0.076 | 0.126 | 0.61 | 0.544 |  |
|  | 2-5 | Intercept | 2.194 | 0.208 | 10.56 | <0.001 |  |
|  |  | GM type (MGM) | 0.294 | 0.139 | 2.12 | 0.034 | 1.34[1.02,1.76] |
|  |  | GM type (MGM.P) | 0.675 | 0.254 | 2.65 | 0.008 | 1.96[1.19,3.23] |
|  |  | GM type (PGM) | 0.049 | 0.142 | 0.34 | 0.731 | 1.05[0.80,1.39] |
|  |  | GM type (PGM.M) | 0.453 | 0.256 | 1.76 | 0.080 | 1.57[0.95,2.60] |
|  |  | GM type (both) | 0.281 | 0.127 | 2.21 | 0.028 | 1.32[1.03,1.70] |
|  |  | Age | 0.378 | 0.057 | 6.58 | <0.001 |  |
|  |  | Mother status (dead) | -0.985 | 0.203 | -4.86 | <0.001 |  |
|  |  | Mother status (censored) | 0.200 | 0.724 | 0.28 | 0.782 |  |
|  | 5-15 | Intercept | 4.517 | 0.260 | 17.40 | <0.001 |  |
|  |  | GM type (MGM) | -0.263 | 0.167 | -1.58 | 0.115 | 0.77[0.56,1.07] |
|  |  | GM type (MGM.P) | 0.331 | 0.299 | 1.11 | 0.268 | 1.39[0.78,2.50] |
|  |  | GM type (PGM) | -0.108 | 0.189 | -0.57 | 0.566 | 0.90[0.62,1.30] |
|  |  | GM type (PGM.M) | 0.027 | 0.287 | 0.09 | 0.926 | 1.03[0.59,1.80] |
|  |  | GM type (both) | 0.120 | 0.187 | 0.64 | 0.522 | 1.13[0.78,1.63] |
|  |  | Age | 0.134 | 0.019 | 7.15 | <0.001 |  |
|  |  | Living siblings | -0.162 | 0.041 | -3.96 | <0.001 |  |
|  |  | Mother status (dead) | -0.873 | 0.182 | -4.80 | <0.001 |  |
|  |  | Mother status (censored) | 0.616 | 1.134 | 0.54 | 0.587 |  |
|  |  |  | Variance | Standard Deviation |  |  |  |
| Random | 0-2 | MGM ID:Mother ID | 0.202 | 0.450 |  |  |  |
|  |  | MGM ID | 0.203 | 0.450 |  |  |  |
|  |  | Birth cohort | 0.031 | 0.177 |  |  |  |
|  | 2-5 | MGM ID:Mother ID | 0.212 | 0.461 |  |  |  |
|  |  | MGM ID | <0.001 | <0.001 |  |  |  |
|  |  | Birth cohort | 0.064 | 0.253 |  |  |  |
|  | 5-15 | MGM ID:Mother ID | 1.021 | 1.011 |  |  |  |
|  |  | MGM ID | 0.113 | 0.336 |  |  |  |
|  |  | Birth cohort | <0.001 | <0.001 |  |  |  |

Fig S1. Birth order-specific probability of reproducing by presence of mother or mother-in-law. Smoothing splines fitted from model-predicted reproduction probabilities using smooth.spline function. Grey line = neither mother nor mother-in-law alive, black = both alive, solid blue = mother alive, dashed blue = mother-in-law alive.
